# Análisis de máxima tensión compresiva en incisivos centrales superiores rehabilitados con postes de fibra de vidrio y tres tipos de coronas. Un estudio con método de elementos finitos

**DOI:** 10.21142/2523-2754-1003-2022-125

**Published:** 2022-09-28

**Authors:** Mayra J. Chávez Vela, Ana I. López Flores

**Affiliations:** 1 División de Rehabilitación Oral de la Universidad Científica del Sur. Lima, Perú. majita.chavez@gmail.com, alopezf@cientifica.edu.pe Universidad Científica del Sur División de Rehabilitación Oral Universidad Científica del Sur Lima Peru majita.chavez@gmail.com alopezf@cientifica.edu.pe

**Keywords:** cerámicas, coronas, análisis de elementos finitos, máxima tensión compresiva, ceramics, crowns, finite element analysis, maximum compressive stress

## Abstract

**Objetivo:**

Evaluar la máxima tensión compresiva en incisivos centrales superiores restaurados con postes de fibra de vidrio y tres tipos de coronas por método de elementos finitos MEF.

**Materiales y métodos:**

El estudio fue un ensayo de laboratorio virtual y descriptivo. Se confeccionaron 3 modelos virtuales mediante el programa SolidWorks 2017, a partir de incisivos centrales superiores rehabilitados con postes de fibra de vidrio y corona metal-cerámica, corona de disilicato de litio monolítica y corona de zirconio-cerámica; luego, estos fueron sometidos a una carga oclusal oblicua de 150 N con una angulación de 45°, distribuida hacia la cara palatina. El análisis de las tensiones se realizó mediante la comparación de las tensiones máximas, mínimas y equivalentes de von Mises.

**Resultados:**

La máxima tensión compresiva se encontró a nivel cervical en la zona vestibular de cada una de las coronas, siendo el diseño 3 (corona de zirconio-cerámica) el que presentó mayor tensión compresiva, con 73,89 MPa, seguido por el diseño 2 (corona de disilicato de litio), con 63,42 MPa, y el diseño 1 (corona metal-cerámica), con 48,4 MPa.

**Conclusión:**

La corona zirconio-cerámica distribuye mejor la tensión a lo largo del diente, ya que, por su rigidez, absorbe las tensiones que se concentran especialmente en cervical, lo que podría indicar que es la opción más apropiada para rehabilitar dientes tratados endodónticamente.

## Abstract (en)

**Objective:**

To evaluate the maximum compressive stress in maxillary central incisors restored with fiberglass posts and three types of crowns by the FEM finite element method.

**Materials and methods:**

The study was a virtual, descriptive, and laboratory trial. Three virtual models were made using the SolidWorks 2017 software from upper central incisors rehabilitated with fiberglass posts and a metal-ceramic crown, a monolithic lithium disilicate crown, and a zirconium-ceramic crown. They were then subjected to an oblique occlusal load of 150N with an angulation of 45°, distributed towards the palatal aspect. The stress analysis proceeded by comparing the maximum, minimum, and equivalent von Mises stresses.

**Results:**

The maximum compressive stress was found at the cervical level in the vestibular area of each of the crowns. Zirconium-ceramic crown (Design 3) was the one with the highest compressive stress with 73.89 MPa, followed by Lithium Disilicate crown (Design 2) with 63.42 MPa and the metal-ceramic crown (Design 1) with 48.4 MPa.

**Conclusion:**

The zirconium-ceramic crown better distributes the stress along the tooth since, due to its rigidity, it absorbs the stresses that are concentrated especially in the cervical area, which could indicate that it is the most appropriate option to rehabilitate endodontically treated teeth.

## INTRODUCCIÓN

La rehabilitación de dientes con tratamientos endodónticos, particularmente en el sector anterior, representa uno de los retos fundamentales que enfrenta el profesional ^(1, 2)^ pues, al haber una serie de opciones científicamente comprobadas, en cierta modo se dificulta la selección de las restauraciones estéticas más apropiadas y naturales ^3^. Además, la estructura dentaria remanente, al ser más frágil que un diente sano, presenta un comportamiento biomecánico diferente ^4^; por lo tanto, el éxito del rendimiento biomecánico está relacionado con múltiples factores que van desde la preservación del tejido dentario y el uso adecuado de materiales biocompatibles con el diente ^2^, hasta las cargas oclusales que este soporta ^5^.

En la actualidad, se indican múltiples materiales para rehabilitación. Uno de ellos es la cerámica feldespática, que muestra notable estética; sin embargo, sus propiedades mecánicas siguen siendo relativamente débiles, por lo cual suele ser utilizado sobre infraestructuras metálicas o cerámicas ^6^. Desde que surgieron los sistemas cerámicos libres de metal, uno de ellos, el disilicato de litio, tiene una resistencia a la flexión de hasta 400 MPa ^7^ y, tanto en el sector anterior como en el posterior presenta una adecuada resistencia a los esfuerzos, por lo que resulta un material idóneo para la rehabilitación de estos casos ^8^.

Hoy en día, las cerámicas de zirconio también están dentro de las indicaciones clínicas, debido a sus excelentes propiedades mecánicas ^9^ y su resistencia a la compresión de 2000 Mpa ^8^, incluso mayor que la de la vitrocerámica de disilicato de litio ^10^. Para la confección de estructuras, está indicado el zirconio 3-mol%-yttria-estabilizado policristales de zirconia tetragonal (3Y-TZP) ^11^, que se caracteriza por presentar una alta resistencia a la flexión (entre 1000 y 1500 MPa); pero debido a la alta opacidad que presenta es necesario el revestimiento con cerámica cuando se requieren restauraciones estéticas ^12^^,^^13^.

El uso de elementos finitos en odontología es cada vez mayor, por tratarse de un método no invasivo ^14^ y versátil, considerado una magnífica herramienta que brinda reproducibilidad y precisión de los resultados. Upadhyaya et al. ^15^ resaltan la importancia del método de elementos finitos porque permite valorar la distribución de tensiones y el rendimiento de las estructuras internas gracias a su capacidad para analizar cuantitativamente el estrés, simulando mediante un modelo matemático el comportamiento de distintos materiales, diseños y técnicas sometidos a diversas cargas, con lo que predice las fallas a largo plazo en ciertas regiones y proporciona datos adicionales para un análisis in vitro ^1^^,^^16^.

Esta investigación buscó evaluar la máxima tensión compresiva en modelos de incisivos centrales superiores rehabilitados con postes de fibra de vidrio y tres tipos de coronas, al ser sometidos a una carga oclusal oblicua de 150 N con una angulación de 45°, distribuida hacia la cara palatina, por medio del MEF. Por lo tanto, los resultados obtenidos en este estudio brindan información importante sobre el comportamiento de ciertos biomateriales utilizados en la rehabilitación con coronas en el sector anterior, lo que sirve como orientación clínica.

## MATERIALES Y MÉTODOS

Se realizó un ensayo de laboratorio virtual de tipo descriptivo y transversal. El programa computarizado que se utilizó fue SolidWorks COSMOSWorks 2017® que, junto con la construcción de los modelos matemáticos, fue manejado por personal capacitado. El grupo de estudio estuvo conformado por 3 modelos virtuales, cada uno con diferente tipo de corona.

### Criterios de selección

Se utilizaron modelos de software SolidWorks 2017 a partir de incisivos centrales superiores rehabilitados con postes de fibra de vidrio, muñón de resina y corona de metal-cerámica, monolítica de disilicato de litio y corona de óxido de zirconio-cerámica. Estos fueron realizados con base en parámetros establecidos, propiedades de los materiales obtenidos a través de módulo elástico, por coeficiente de Poisson.

El proceso de simulación constó de 3 pasos: 

### Primer paso

Construcción de los modelos geométricos, para lo que se creó un modelo virtual base de incisivo central superior con longitud total de 23 mm; la corona midió 9 mm de longitud por 8 mm de amplitud; y la raíz midió 14 mm de longitud más 2 mm de estructura dentaria para el efecto férula ^17^. Luego, se replicaron 3 modelos con las respectivas coronas en 3D. 

**Figura 1:**
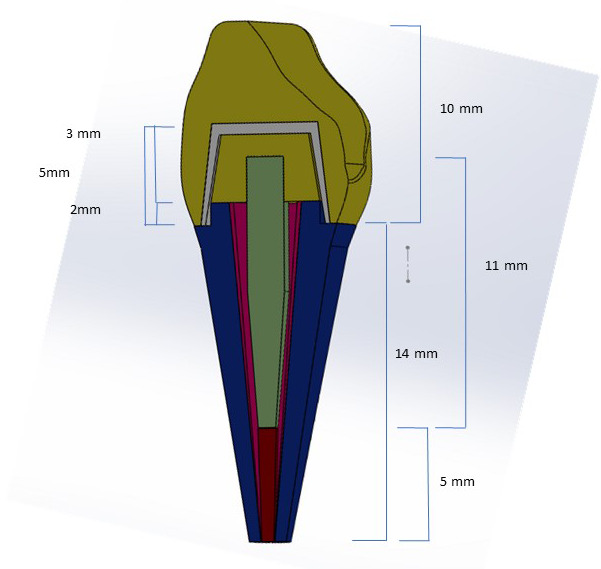
Modelo virtual base incisivo central superior

Para efectos de complemento del estudio, se consideró diseñar las siguientes estructuras: bloque de hueso y ligamento periodontal para fijar el modelo de incisivo virtual; diseño de 5 mm de gutapercha en el conducto según literatura ^5^ y 9 mm para el poste de fibra de vidrio de doble conicidad (1,6 mm de diámetro en incisal y 0,85 mm en apical) ^2^^,^^4^. El muñón se construyó con una longitud de 5 mm en altura inciso-gingival y estuvo compuesto por 3 mm de resina junto a los 2 mm del remanente dentario (efecto férula) ^17^ (Fig. 1).

Simultáneamente se confeccionaron 3 modelos de coronas virtuales teniendo en cuenta la altura y amplitud de la corona del modelo virtual base:

• Modelo 1. Corona metal cerámica, cofia de 0,5 mm, cerámica de 1,0 mm, en zona incisal 2 mm (2, 18). 

• Modelo 2. Corona monolítica de disilicato de litio de 1,5 mm ^19^.

• Modelo 3. Corona óxido de zirconio-cerámica, cofia de 0,5 mm ^20^, cerámica 1,0 mm, 1,5 mm en zona incisal ^1^. 

Posteriormente, se anexó cada corona virtual a cada modelo virtual base de incisivo central superior para conformar los 3 modelos finales a los cuales se les aplicaron las fuerzas. 

Las propiedades mecánicas de los elementos que componen los modelos matemáticos fueron obtenidas de estudios previos de la literatura (Tabla 1).

**Tabla 1:** Propiedades mecánicas de los materiales

Material	Módulo elasticidad (GPa)*	Coeficiente de Poisson
Dentina	18.6	0.31 (2)
Ligamento periodontal	68.9	0.45 (16)
Poste fibra de vidrio Whitepost (FGM)	45	0.25 (21)
Muñón de resina	12	0.30 (2,4)
Cofia metal	95	0.33 (21)
Cerámica vítrea de nano fluorapatita	69	0,28 (2, 22)
Disilicato litio	95	0,26 (2, 23, 24)
Cerámica feldespática	68.7	0,20 (24)
Cofia de óxido de Zirconio	210	0,26 (12,25,26)

*GPa (Gigapascales)

### Segundo paso

**Figura 2:**
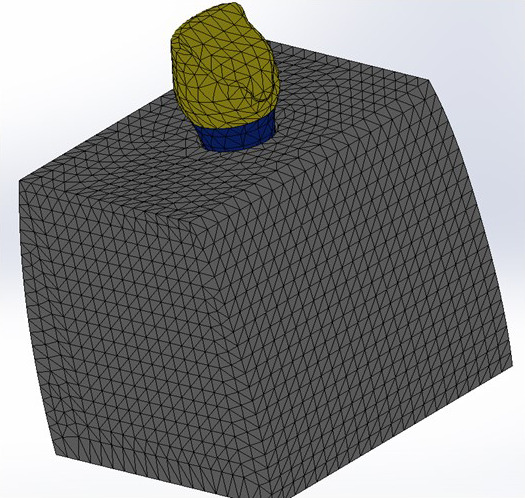
Mallado

Elaboración del mallado, el cual consistió en la utilización de una malla fina para cada uno de los 3 diseños (Fig. 2).

### Tercer paso

Aplicación de fuerzas. Se aplicó a los modelos una carga de 150 N ^(5, 27, 28)^ uniformemente distribuida hacia la cara palatina, con un ángulo de 45° ^2^^,^^28^^-^^29^, mediante una simulación de la carga oblicua que se genera durante los movimientos masticatorios. Posteriormente, se evaluaron las tensiones compresivas ^29^. 

Para cada modelo matemático, se calcularon los esfuerzos Von Mises, los esfuerzos máximos y mínimos principales.

## RESULTADOS

Se encontró que el modelo 3, corona de zirconio-cerámica, presentó una mayor tensión, con 73.89 MPa, a diferencia del modelo 2, con 63.42 MPa, y el modelo 1, con 48.4 MPa (Tabla 2).

**Figura 3:**
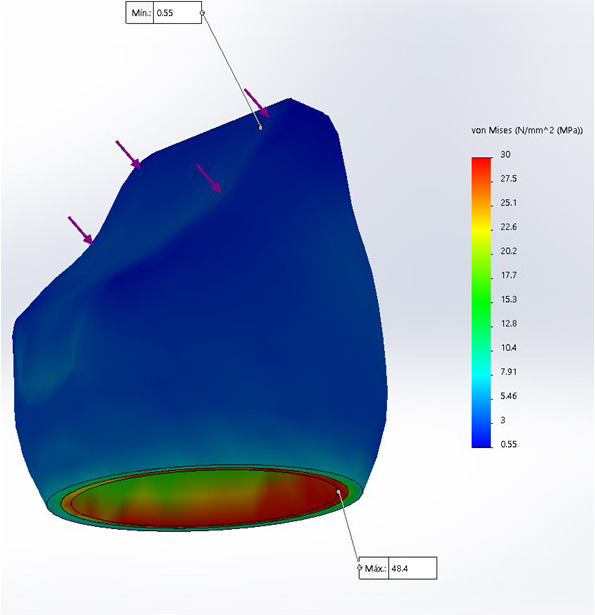
Análisis de Von Mises para tensión compresiva sobre corona metal-cerámica

**Tabla 2:** Tensión compresiva sobre coronas (MPa)*

Modelos	Máxima tensión	Mínima tensión
Corona metal cerámica Modelo 1	48,4	0,55
Corona disilicato monolítico Modelo 2	63,42	0,25
Corona zirconio cerámica Modelo 3	73,89	0,60

*MPa: megapascales

Además, en los tres diseños, el análisis de Von Mises para la máxima tensión compresiva se encontró a nivel cervical en la zona vestibular de las 3 coronas.

**Figura 4:**
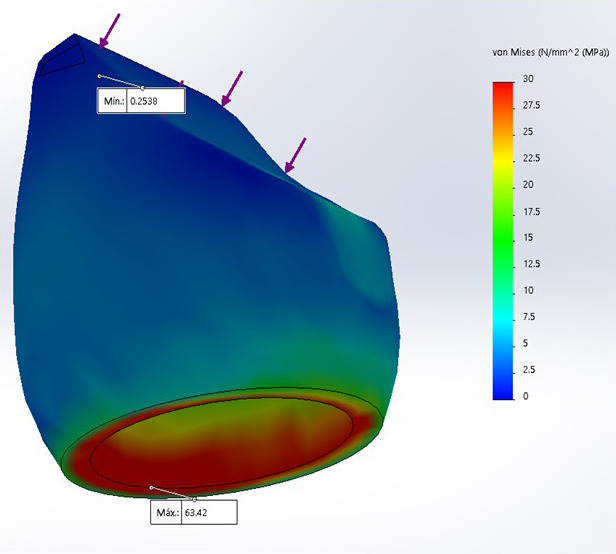
Análisis de Von Mises para tensión compresiva sobre corona disilicato de litio monolítico

En el análisis de la tensión compresiva sobre corona metal-cerámica, se encontró un valor de tensión máxima de 48,4 MPa para metal y 20,32 MPa para cerámica.

**Figura 5:**
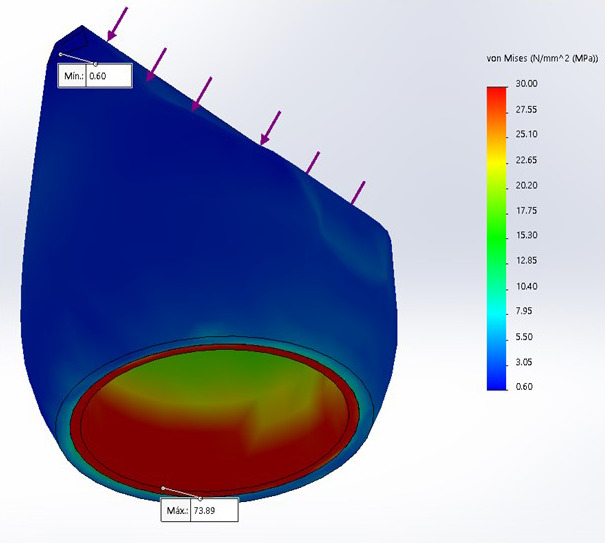
Análisis de Von Mises para tensión compresiva sobre corona de zirconio-cerámica

En el análisis de la tensión compresiva sobre el modelo 2, corona de disilicato de litio monolítica, se encontró valor de tensión máxima de 63,42 MPa.

En el análisis de la tensión compresiva sobre el modelo 3, corona de zirconio-cerámica, se encontró valor de tensión máxima de 73,89 MPa.

Al analizar la tensión compresiva en el muñón y raíz con espigo (bloque), la máxima tensión se observó en la zona cervical de los tres modelos, siendo el modelo 2 el que mostró mayor tensión, con 28,55 MPa, seguido por el modelo 1, con 22,37 MPa, y el modelo 3, con 21,09 MPa (Tabla 3).

**Tabla 3:** Tensión compresiva en muñón y raíz con espigo (MPa)*

Modelos	Máxima tensión	Mínima tensión
Corona metal cerámica modelo 1	22,37	2,58
Corona disilicato monolítico modelo 2	28,55	2,77
Corona zirconio cerámica modelo 3	21,09	0

*MPa: megapascales

## DISCUSIÓN

En este estudio se planteó evaluar la máxima tensión compresiva en 3 modelos de incisivos centrales superiores con poste de fibra de vidrio y rehabilitados cada uno con un tipo de corona (metal-cerámica, disilicato monolítico, óxido de zirconio-cerámica), a través del MEF, para ser sometidos a una carga oclusal oblicua de 150 N, teniendo en cuenta una angulación de 45° distribuida hacia la cara palatina.

El MEF es y ha sido utilizado para tratar de comprender cómo es la distribución de los esfuerzos y, a la vez, entender el comportamiento biomecánico, en este caso, de los incisivos tratados endodónticamente, restaurados con postes y prótesis fija cuando están en función.

Al realizar el análisis de Von Mises en los tres diseños, la máxima tensión compresiva se encontró a nivel cervical en la zona vestibular de las coronas, siendo el modelo 3 (óxido de zirconio-cerámica) el que presentó una mayor tensión máxima, con 73,89 MPa, a diferencia del modelo 2 (disilicato monolítico), con 63,42 MPa, y el modelo 1 (metal-cerámica), con 48,4 MPa. Estos resultados coinciden con los obtenidos por Sánchez et al. ^18^ y Nahar et al. ^4^, quienes realizaron estudios sobre incisivos centrales con diferentes postes y coronas de metal-cerámica, en los que, a pesar de que cada estudio aplicó diferentes fuerzas (200 y 100 N, respectivamente), pero con la misma angulación usada en este estudio, obtuvieron que las máximas tensiones compresivas también se presentaron en la cara vestibular del incisivo central. Además, también concuerda con Alfaro et al. ^21^ quienes, a pesar de haber trabajado en premolares, mencionan que la máxima tensión compresiva se observó en la zona vestibular de los modelos estudiados.

El modelo que tuvo mejor rendimiento con respecto a la aplicación de tensión compresiva fue el modelo 3 (zirconio-cerámica) con 73,89 MPa; este resultado difiere del estudio de Moris ^1^^)^ que, aunque trabajó en caninos, concluye que la corona metal-cerámica muestra un mejor rendimiento a pesar de su estética desfavorable, lo cual sugiere que es un material apropiado para la restauración de dientes tratados endodónticamente.

De los tres modelos analizados en el estudio, se obtuvo que, después del modelo 3, el siguiente que distribuye mejor la tensión compresiva fue el modelo 2 (disilicato monolítico), con 63,42 MPa. Estos datos tienen cierta similitud con el estudio de Vargas ^24^, quien, al evaluar la tensión compresiva en premolares mediante el método de elementos finitos, demostró también que la corona de disilicato de litio distribuye mejor la tensión.

El estudio de Sorrentino et al. ^30^, que evaluó la distribución de los esfuerzos en todo el sistema conformado por el poste, el muñón y la corona, respalda la importancia de un material con un módulo de elasticidad más alto para la corona, que cubra todo el sistema y así evite mayores esfuerzos en todo el diente. Los hallazgos de este autor coinciden con el presente estudio, donde se encontró que el modelo 3 (zirconio-cerámica), al presentar un mayor módulo de elasticidad y, por tanto, al tener mayor rigidez que las coronas de los otros dos modelos, también absorbió máxima tensión, con 73,89 Mpa, y transmite un menor esfuerzo, de 21,09 Mpa, a la raíz con espigo y muñón en bloque, es decir, a todo lo largo de la raíz.

Además, es importante resaltar que la razón de que la mayor tensión compresiva se haya encontrado en la zona cervical de los modelos del estudio realizado. Esto se puede deber a la discontinuidad de los materiales en esa zona por sus diferentes propiedades mecánicas, teoría que es respaldada por Sorrentino et al. ^30^, quienes mencionan que las interfaces de materiales con diferentes módulos de elasticidad representan el eslabón débil de los sistemas de restauración y, por lo tanto, las tensiones se concentran en esa zona.

A pesar de que en el estudio se utilizó una carga estática y no cíclica sobre los modelos, como la que se produce en la cavidad oral, el MEF permitió obtener los resultados más próximos a lo que podría en una situación clínica, y así tomar las decisiones más adecuadas.

## CONCLUSIONES

La corona zirconio-cerámica distribuye mejor la tensión a lo largo del diente, ya que su rigidez absorbe las tensiones que se concentran especialmente en cervical, lo que podría indicar que es la opción más apropiada para rehabilitar dientes tratados endodónticamente.

Existen mínimas diferencias, pero importantes, en los resultados de análisis de máxima tensión compresiva de los tres modelos estudiados. 

## Fuente de financiamiento:

Autofinanciado
